# Noninvasive inter- and intrafractional motion control in ultrahypofractionated radiation therapy of prostate cancer using RayPilot HypoCath™—a substitute for gold fiducial-based IGRT?

**DOI:** 10.1007/s00066-023-02125-2

**Published:** 2023-08-25

**Authors:** Johannes Berchtold, Carmen Winkler, Josef Karner, Michael Groher, Christoph Gaisberger, Felix Sedlmayer, Frank Wolf

**Affiliations:** 1 Dpt. of Radiation Oncology, Paracelsus Medical University of Salzburg, Müllner Hauptstraße 48, 5020 Salzburg, Austria; 2 Paracelsus Medical University of Salzburg, Stubergasse 21, 5020 Salzburg, Austria

**Keywords:** Noninvasive tracking, Prostate motion management, Image-guidance alternatives, Radiosurgery, Margins

## Abstract

**Purpose:**

In ultrahypofractionated radiation concepts, managing of intrafractional motion is mandatory because tighter margins are used and random errors resulting from prostate movement are not averaged out over a large number of fractions. Noninvasive live monitoring of prostate movement is a desirable asset for LINAC-based prostate stereotactic body radiation therapy (SBRT).

**Methods:**

We prospectively analyzed a novel live tracking device (RayPilot HypoCath™; Micropos Medical AB, Gothenburg, Sweden) where a transmitter is noninvasively positioned in the prostatic urethra using a Foley catheter in 12 patients undergoing ultrahypofractionated intensity-modulated radiation therapy (IMRT) of the prostate. Gold fiducials (Innovative Technology Völp, Innsbruck, Austria) were implanted to allow comparison of accuracy and positional stability of the HypoCath system and its ability to be used as a standalone IGRT method. Spatial stability of the transponder was assessed by analyzing transmitter movement in relation to gold markers (GM) in superimposed kV image pairs. Inter- and intrafractional prostate movement and the impact of its correction were analyzed.

**Results:**

A total of 64 fractions were analyzed. The average resulting deviation vector compared to the GM-based position was 1.2 mm and 0.7 mm for inter- and intrafractional motion, respectively. The mean intrafractional displacement vector of the prostate was 1.9 mm. Table readjustment due to exceeding the threshold of 3 mm was required in 18.8% of fractions. Repositioning reduced the time spent outside the 3‑mm margin from 7.9% to 3.8% of beam-on time. However, for individual patients, the time spent outside the 3‑mm margin was reduced from to 49% to 19%.

**Conclusion:**

the HypoCath system allows highly accurate and robust intrafractional motion monitoring. In conjunction with cone beam CT (CBCT) for initial patient setup, it could be used as a standalone image-guided radiation therapy (IGRT) system.

## Introduction

Primary external beam radiation therapy (EBRT) is an equivalent treatment alternative to surgery for localized prostate cancer of all risk groups. In addition, it is the preferred treatment of the primary in de-novo metastasized prostate cancer and has been shown to improve progression-free (PFS) as well as overall survival (OS) in low-volume disease [1, 2]. Ultrahypofractionated regimens (UHFX), also known as stereotactic body radiation therapy (SBRT), have shown compelling results in clinical trials comparing UHFX with normo- or moderately hypofractionated radiation therapy [3–5] and have become a treatment alternative which is supported by international guidelines such as those from the NCCN [6].

The broader use of UHFX in primary prostate cancer has increased interest in an image-guided radiation therapy (IGRT) system which allows both inter- and intrafractional movement control. The latter is mandatory because tighter margins need to be used and random errors resulting from prostate movement are not “averaged out” over a large number of fractions [7].

Prostate movement varies significantly between patients and has been shown to increase as a function of elapsed time [8], underlining the importance of motion control during longer fractions (and of aiming for the shortest possible treatment times).

Commonly available IGRT protocols such as cone beam CT (CBCT) and planar X‑ray imaging (kV-IGRT) using the built-in kV panel of modern linear accelerators can be used to account for intrafractional motion [4]. However, they do not allow for continuous monitoring of prostate position, and radiation needs to be halted during image acquisition, which is time consuming and exposes the patient to additional dose.

These shortcomings are addressed by IGRT technologies using electromagnetic transponder beacons which are transiently implanted into the prostate and whose position is recorded in real time via a detector plate sitting on top of the treatment table. These have been used with success [9, 10] but require additional IGRT methods due to interfractional transmitter positional instability and involve an implantation and explantation procedure requiring general anesthesia and relevant patient discomfort invoked by the suture.

Alternatively, permanent transperineal implantation of electromagnetic transponder beacons, e.g., Calypso® (Calypso Medical Technologies, Seattle, WA, USA), has been used [8, 11, 12]. This, however, carries the obvious drawbacks of a transperineal invasive procedure: patient discomfort, requirement of anesthesia or pain medication, prophylactic use of antibiotics, and discontinuation of oral anticoagulation.

**Fig. 1:**
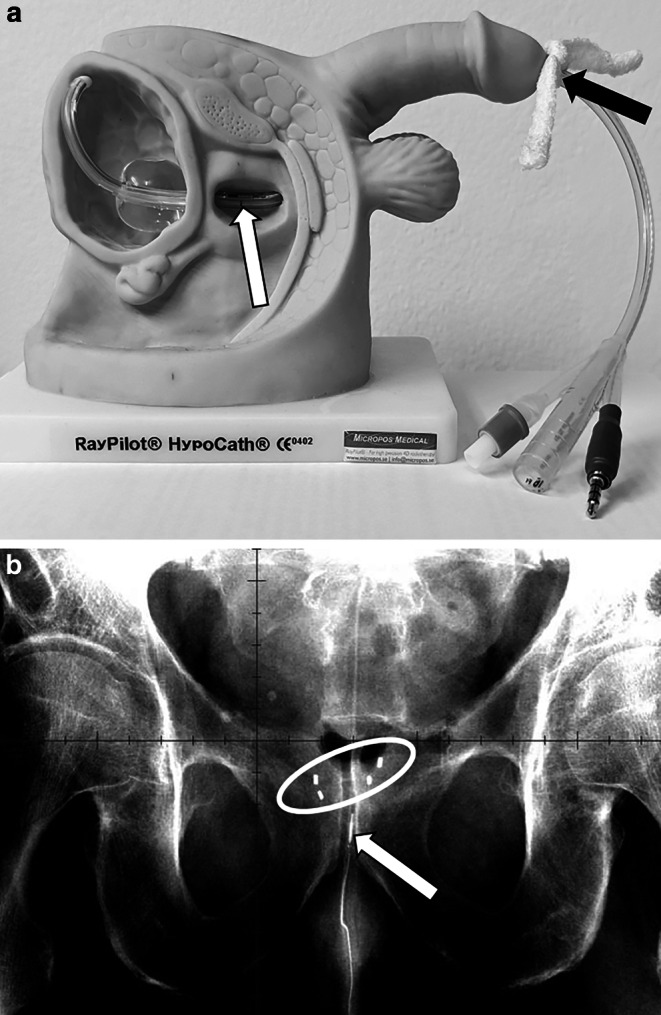
**a** Schematic view of the RayPilot HypoCath™ (Micropos Medical AB) in situ. *White arrow* indicates the position of the transmitter. A constant slight pull of the HypoCath catheter was established by tying a gauze bandage (*black arrow*) and pushing it firmly towards the glans penis. **b** 180° kV image as used for IGRT of an exemplary patient with gold fiducials (*circle*) and HypoCath transmitter (*arrow*) in situ

RayPilot HypoCath™ (Micropos Medical AB, Gothenburg, Sweden) is a novel IGRT system where the transmitter is placed into the prostatic urethra in a noninvasive manner via a urine catheter resembling a regular 14 mm Foley catheter with co-axial wiring and a connector which attaches to the detector plate (Fig. 1). The blocking balloon inside the bladder ensures safe and reproducible positioning of the transmitter. The catheter is placed prior to the planning CT and remains in place during the whole treatment period.

Here we report on our clinical experience in 12 patients with metastatic prostate cancer receiving UHFX with 6 × 6 Gy and provide a thorough analysis of the absolute benefit in terms of fraction duration, prostate motion, and the stability of the transmitter in relation to gold markers (GM) during and between fractions. The latter allows assessment of its possible future use as a standalone IGRT system that reliably tracks both inter- and intrafractional motion without the need of additional X‑ray-based imaging.

## Materials and methods

### Patient cohort and treatment planning

All patients had primary metastasized prostate cancer and were treated for the primary according to the recommendation of our local tumor board. Patients received ultrahypofractionated radiation with 6 × 6 Gy to the prostate three times a week using 15-MV volumetric arc therapy (VMAT) with two arcs on a Elekta Synergy LINAC (Elekta Solutions AB, Stockholm, Sweden). Prior to radiation, patients received four gold marker implants. In 2 patients, a gel spacer SpaceOAR™ System (Augmenix Inc., Waltham, MA, USA) was implanted in the same session as described previously [13, 14].

A planning MRI was performed without the catheter due to its magnetic properties.

The RayPilot HypoCath™ catheter (Micropos Medical AB) was placed the following day prior to the planning CT according to the manufacturer’s protocol. Radiation was commenced the same or the following day.

### Patient setup and intrafractional motion control

Prior to the CT and each fraction, the bladder was filled with 170–200 ml saline and the rectum was emptied using mild laxatives or clyster. In order to establish a reproducible pull on the catheter, a tight knot of gauze bandage was tied around the catheter and firmly pushed toward the glans penis to create a counter pressure (see Fig. 1a).

The RayPilot system was set to a threshold of 3 mm in all directions. After completion of setup, prostate movement was monitored for 1–3 min until initial movement had settled. Then, two kV images at 180° and 130° (for best visibility of the gold markers) were acquired (Fig. 1b for an exemplary 180° kV image) and the RayPilot system nulled synchronously with acquisition of the fist image. The patient was matched based on the gold markers and radiation started. If the prostate moved outside the 3‑mm threshold, radiation was halted. If the position remained outside the 3 mm margin for an extended period of time (around 20 s), kV imaging was reinitiated and the RayPilot system nulled as described.

### Data analysis

During all fractions, prostate coordinates as recorded by the HypoCath transmitter were stored in an SQL database at 1‑s intervals by the RayPilot software (Micropos Medical AB). Further data analysis and graphical representation was performed using an in-house developed Python script.

To allow comparative analysis with non-HypoCath-corrected radiation delivery, a hypothetical, simulated standard fraction duration without HypoCath correction (including time for initial setup and delivery time of arcs 1 and 2 with a short stop in between) was generated using a Python script.

### Transmitter stability

Axial movement of the catheter was assessed by matching 180°kV images based on gold markers and calculating the offset of the transmitter. Since the prostatic urethra runs through the prostate in a nearly vertical fashion, 2D projection to the coronary 180° kV images is well suited to detect axial movement of the catheter within the urethra.

For interfractional movement, the first images of each fraction were analyzed. For intrafractional stability, only images within one fraction were compared.

## Results

### Fraction duration/additional time required

A total of 64 fractions applied to 12 different patients treated between March 2021 and July 2022 were recorded and analyzed. In 8 fractions the simultaneous nulling of the HypoCath system with the kV-IGRT was not recorded with the correct timestamp. Those fractions were therefore excluded from the analysis.

All patients had metastasized, low-volume disease according to the CHAARTED criteria. Median age was 71.5 years. All patients had received androgen deprivation therapy (ADT), median PSA was 0.22 ng/ml.

The RayPilot monitoring led to 21 treatment interruptions due to violation of the 3‑mm threshold (on average 0.3 interruptions per fraction). For individual patients, radiation was halted up to five times per fraction, 8 of 12 patients had at least one interruption during the course of their treatment.

The mean total fraction duration starting with kV image acquisition for IGRT and ending with delivery of the last monitor unit was 9 min, of which 3:15 min was required for initial IGRT (acquisition, matching, and readjustment of table position), 0:30 min for gantry rotation between the first and second arcs, 1:15 min for additional interruptions and IGRT triggered by the HypoCath system, and 4 min were actual beam-on time. The relative additional time consumed by HypoCath-related imaging and setup was 16% compared to a hypothetical standard fraction of 7.45 min.

### Prostate motion with and without correction

Considering only beam-on time, the mean shift of the mean resulting displacement vector of the prostate was 1.7 ± 0.4 mm in corrected fractions. In a hypothetical simulated uncorrected fraction, the mean shift was 1.9 ± 0.4 mm.

However, if only fractions were considered where a correction was performed, the mean shift of the resulting prostate vector was 2.3 ± 0.9 mm and 3.1 ± 0.8 mm for the corrected and the hypothetical uncorrected session, respectively, corresponding to a benefit of 0.8 mm. This translates to a considerable reduction of time spent outside a 3-, 4‑, and 5 mm margin (Table 1).

**Table 1:** Time spent outside a 3-, 4‑, or 5‑mm margin during beam-on time with and without repositioning after IGRT initiation by the HypoCath system for fractions with at least one interruption

Time spent outside	3 mm	4 mm	5 mm
Without interaction, in %	34 (8)	9 (2)	3 (1)
With interaction, in %	15 (4)	2 (0)	1 (0)

Numbers in parenthesis show all fractions

Prostate motion correlated with time in an almost linear fashion. The average speed of prostate motion for all patients was 1 mm/5 min. The mean standard deviation of the difference between the linear fit and the real prostate motion was 0.4 mm. Interrupting the treatment for repositioning took 5:08 min on average before treatment could be continued. This interruption corresponds to a mean prostate shift of 1 ± 0.4 mm for this timeframe.

There was great heterogeneity among patients in terms of prostate stability, with some patients featuring only small prostate deviations with no need for intervention in any of the fractions while others had highly mobile prostates with up to five interventions in one fraction.

**Fig. 2:**
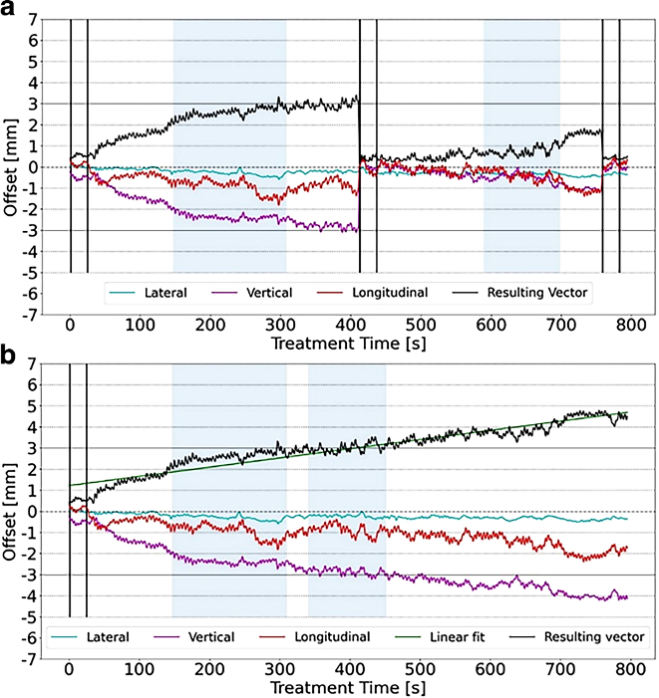
Prostate intrafractional movement. **a** Prostate movement in lateral, vertical, and longitudinal axes of a representative fraction of an individual patient with HypoCath-initiated correction. *Vertical black lines* indicate kV-IGRT, repositioning of the patient, and nulling of the HypoCath system, *blue shaded areas* indicate beam-on time. **b** Hypothetical fraction of the same patient without repositioning. The *green line* indicates the linear fit of the resulting prostate motion

In order to appreciate the impact of intrafractional control in such patients, we analyzed the time spent within the 3‑mm threshold of the 3 patients with the largest displacement vector resulting in a decrease of 49% to 19%, from 32% to 21%, and from 31% to 20% (average reduction of 44%) of the time spent within 3 mm (see Fig. 2a, b for an exemplary patient).

### Transmitter stability in relation to gold fiducials

In order to assess whether the transmitter remained stationary within the prostate throughout and between fractions, we assessed the relative position of the transmitter in relation to the gold fiducials.

**Fig. 3:**
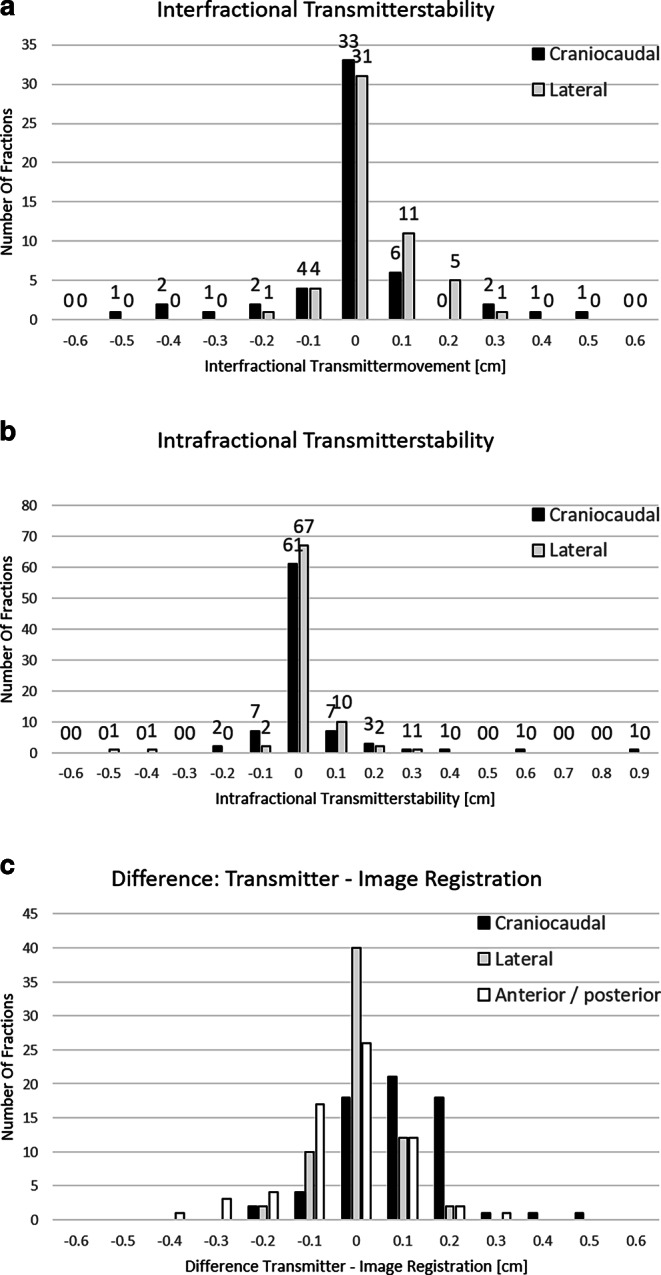
Transmitter stability in coronal plane in relation to gold fiducials. Interfractional (**a**) and intrafractional (**b**) offsets in cranial (*black bars*) and lateral (*grey bars*) direction. **c** Offsets of HypoCath-generated values from kV-IGRT-derived values in longitudinal (*black bars*), lateral (*grey bars*), and anteroposterior (*dotted bars*) directions

Comparing image pairs of consecutive fractions or within one fraction allowed us to separately analyze inter- and intrafractional stability of the transmitter in relation to the GM in the coronary plane. The mean absolute deviation was 0.9 ± 1.5 mm in cranial/caudal and 0.5 ± 0.7 mm in left/right, the resulting mean displacement vector was 1.2 mm ± 1.5 mm (Fig. 3a). The absolute deviation was 0.5 ± 1.3 mm in cranial/caudal and 0.3 ± 0.8 mm in left/right, the resulting mean displacement vector was 0.7 mm ± 1.5 mm (Fig. 3b).

In one of the 64 analyzed fractions, abnormal incongruity was detected with an offset of 0.54 cm and 0.92 cm in two subsequent kV images acquired after 440 s and 790 s of treatment time, respectively, indicating movement of the transmitter in the urethra. That particular patient had a highly mobile prostate and had reported urge and pain during the retrograde filling of the bladder. Target volume coverage and OAR dose were not significantly affected.

### Correlation of GM-based kV-IGRT-derived offset values with synchronized HypoCath values

During patient treatment, offset values based on GM-matched kV-IGRT were compared with the synchronized values of the HypoCath system at the exact timepoint the first kV image was acquired.

The mean absolute difference of HypoCath-predicted and kV image-calculated position corrections were 1.1 mm ± 0.9 mm, 0.4 mm ± 0.6 mm, and 0.9 ± 0.9 mm in craniocaudal, lateral, and anterior–posterior directions, respectively (Fig. 3c).

### Impact of HypoCath threshold on PTV margin

Most ultrahypofractionation studies used a posterior PTV margin in the range of 3–5 mm. We have retrospectively calculated which PTV margins of our cohort could safely be used if the RayPilot system is set to a threshold of 3 mm using the commonly used Van Herk margin recipe $M=2.5\Upsigma +0.7\sigma$ [15, 16], where Σ is derived by calculating the standard deviation (SD) of the mean of the daily shifts of each patient, while *σ* is the root mean square of the SD of the daily measurements of each patient. This recipe ensures a minimum dose to the CTVs of 95% for 90% of the patient population.

With a threshold of 3 mm for our cohort, a PTV margin of 2.9/2.4/3.2 mm was derived in craniocaudal, lateral, and anterior–posterior directions, respectively.

### Catheter-related toxicity and patient tolerance

Once in place, the HypoCath catheter was tolerated well by all patients. The bladder filling caused slight discomfort for some. In such cases, the filling duration was extended and the filling volume was reduced from 180 ml to 125 ml.

In one patient (data not shown) catheter placement was not tolerated and had to be aborted due to pain. That patient underwent regular moderately hypofractionated therapy and was not included in the cohort. One patient developed a urinary tract infection 1 week after removal of the catheter necessitating antibiotic treatment. In one patient, the blocking balloon was misplaced in the urethra and caused a minor urethral laceration with minor bleeding and pain. The procedure was repeated the same day without complications.

## Discussion

UHFX of primary and metastatic prostate cancer is supported by NCCN guidelines and is becoming an increasingly used alternative to normo- and moderately fractionated regimens due to its obvious advantages in terms of time, resources, and patient comfort. In addition, dose-escalated regimens with a focal boost to the intraprostatic dominant lesions have shown promising results [17]. These new concepts are, however, highly sensitive to movement of the prostate and call for a reliable and easy to use tool to ensure intrafractional control.

The HypoCath system claims to be such a tool and could—in theory—be used for both, setup and intrafractional control, thus eliminating the need for (invasive) gold marker implantation.

Panizza et al. have recently reported first clinical experiences with the RayPilot system in 13 patients [18], confirming its feasibility in LINAC-based SBRT of the prostate. Their observations on prostate motion are in good concordance with our data. The lack of gold markers did not allow any assessment of the stability of the transmitter within the prostate.

Gold fiducial-based IGRT has become the gold standard for prostate IGRT. However, gold marker implantation is an invasive procedure and comes with possible complications such as bleeding, urinary tract infection and patient discomfort. We have therefore analyzed whether patient setup using HypoCath coordinates alone is comparable in terms of precision and reliability and could make gold marker implantation dispensable. With an average intrafractional offset of the transmitter to the gold markers of 0.7 mm, our results show that the position of the transmitter is very stable in relation to the gold markers. It needs to be emphasized that this offset represents an overestimation, since it includes inaccuracies resulting from matching and changes in the geometry of gold markers due to rotation. This becomes evident when looking at movements of the transmitter relative to the gold markers in the lateral direction of up to 5 mm (mean 0.3 mm), which is obviously a result of misprojection due to rotation since the urethra is unlikely to move inside the prostate gland.

We believe that a reproducible and constant pull on the catheter is crucial for axial stability of the transmitter within the urethra. We found that using gauze bandage knotted around the catheter and pushed against the glans penis was easy to use and generated a constant pull which was independent from movement of the patient’s legs.

In its current form, however, the RayPilot system can detect prostate movement but relies on further independent imaging in order to correct the table position. The time consumption of such imaging was significant, accounting for approximately 5 min per interruption to the treatment time. In some patients, that added up to 8 min, accounting for 62% of the treatment time, during which the prostate is subjected to further movement. Setting the threshold tighter, e.g., to 2 mm will enhance the frequency of interruptions—and thus the time designated to imaging—further. Therefore, direct control of the table position by the HypoCath system would be highly desirable, mitigating the need for extra imaging and enhancing both precision and time consumption. In addition, a flattening filter-free setup could reduce beam-on time by about 65% compared to a 15-MV setup (delivery time estimated by RayStation software, RaySearch Laboratories, Sweden; data not shown) and thus lower the number of beam interruptions further.

We have shown that the HypoCath transmitter is stable in relation to gold markers and that the RayPilot system can be used as a standalone IGRT method using a PTV margin of 3 mm in the posterior direction. For initial patient setup, however, additional IGRT such as CBCT is needed for safety reasons since minor movement of the catheter between fractions would go undetected and would introduce a systematic error.

An alternative to gold marker- or HypoCath-based real-time imaging is MR-guided IGRT, which allows direct monitoring of prostate motion without the use of fiducial markers and which has been shown to reduce acute toxicity in a randomized trial [19]. However, broad implementation is hampered by its immense costs and limited throughput capabilities, especially when compared to the RayPilot system, which is both relatively cheap and fast.

The limitations of our study are the limited number of patients and its non-randomized fashion. Acute and long-term toxicity of the RayPilot system compared to other real-time IGRT methods will have to be assessed in future studies.

## Conclusion

The RayPilot system is an innovative new tool able to reliably control intrafractional motion. For highly dose-escalated treatments or focal boost regimens where precision and very tight margins are mandatory, HypoCath is an alternative to MR-guided or gold fiducial-based real-time imaging.
